# The association between social capital and mental health among older people living with HIV: a systematic review

**DOI:** 10.3389/fpubh.2026.1827140

**Published:** 2026-10-05

**Authors:** Hongyu Xia, Xinjing Cao, Ying He, Le Yang, Qi Yu

**Affiliations:** 1 Shanxi Provincial Key Laboratory of Artificial Intelligence-Enhanced Clinical Decision Support, Shanxi Medical University, Taiyuan, Shanxi, China; 2 School of Management, Shanxi Medical University, Taiyuan, Shanxi, China

**Keywords:** HIV, mental health, older people, social capital, social support, systematic review

## Abstract

**Background:**

Older people living with human immunodeficiency virus (HIV) constitute a vulnerable group in society and are at high risk of developing mental health problems. Although strengthening social capital is a common recommendation to reduce such mental health risks, no systematic review has examined the link between social capital and mental health among older people living with HIV.

**Methods:**

We conducted a systematic review of peer-reviewed studies examining the association between social capital and mental health among adults aged ≥50 years living with HIV. Seven databases were searched up to November 28, 2025. Studies were eligible if they quantitatively assessed at least one dimension of social capital in relation to a mental health outcome in this population. Two reviewers independently screened studies and assessed risk of bias using the Newcastle-Ottawa Scale. Owing to substantial heterogeneity, findings were synthesized narratively.

**Results:**

Among the 9,060 articles identified, 10 articles were included in this systematic review. Nine of 10 studies measured social support. Trust and civic participation were only partially captured within a broader social capital instrument in two reports from the same cohort, and reciprocity and bonding/bridging/linking capital were not measured. The instruments used to measure both social capital and mental health differed substantially across studies, and the dimensions assessed were inconsistently operationalized, which limited direct comparability of findings. Among studies measuring social support, higher levels were generally associated with lower depressive symptoms and better mental well-being, but the strength and significance of associations varied. Two reports from the same cohort assessed community/society-level social capital, reporting mixed associations. Evidence for outcomes other than depressive symptoms was sparse.

**Conclusion:**

Overall, research attention to social capital and mental health among older people living with HIV remains insufficient. Research in this field has overwhelmingly focused on social support, leaving broader dimensions of social capital largely unexamined. The available evidence suggests a beneficial association between social support and mental health, and heterogeneity in measurement instruments and operational definitions further limits comparability across studies. Future studies should devote greater attention to social capital and mental health in this population by adopting standardized and multidimensional social capital measures, and using longitudinal designs to clarify directionality.

**Systematic review registration:**

The protocol for this systematic review was registered in PROSPERO. Unique Identifier: CRD42024537839, https://www.crd.york.ac.uk/prospero/display_record.php?RecordID=537839.

## Introduction

In recent years, the global trend of population aging has intensified, leading to an increase in HIV infections among older people. The aging of people living with HIV has garnered significant attention worldwide (1). According to a recent study, there were an estimated 77,327 new cases of HIV infection among individuals aged 60–89 years worldwide in 2019 (2). The age-standardized rate of new HIV infections in this group is 7.6 cases per 100,000 people, highlighting the challenges faced by the older people in sexually transmitted disease (STD) prevention and control. Between 1990 and 2019, the global prevalence of HIV in the 60–89-year-old population significantly increased in Eastern Europe, Central Asia, and the high-income Asia–Pacific region, with average annual growth rates of 17.84, 14.26, and 7.52%, respectively (2).

Extensive literature suggests that older people living with HIV (OPLWH) face complex psychosocial challenges due to the dual burdens of HIV infection and aging. These challenges lead to a greater risk of mental health problems, including stigmatization, loss of friends, disengagement from social networks, and adverse effects of HIV and antiretroviral therapy on the normal aging process (3). Depression and anxiety are the most prevalent mental health problems among older people living with HIV. A recent systematic review and meta-analysis of 12 observational studies including 5,868 older people living with HIV estimated a pooled prevalence of depression of 28% (95% CI 22–33%), with prevalence ranging from 14.5% in the Netherlands to 42.4% in South Africa (4). Similarly, a study of 72 older people living with HIV in Brazil found that 27.7% had a diagnosis of major depression (5).

Some studies suggest that social capital may be an important determinant of mental health among older people (6, 7). Conceptually, social capital encompasses resources embedded in social relationships and community life, including social support, trust, reciprocity, civic engagement, and social cohesion (8–11). It is typically categorized into structural dimensions (people’s behaviors, such as participation and connections within social networks) and cognitive dimensions (people’s feelings, such as trust and perceived support), as well as three forms: bonding, bridging, and linking-type (12, 13). These distinctions are crucial because different dimensions of social capital may influence mental health through distinct mechanisms. For example, receiving support may buffer the immediate effects of stress, while trust in institutions or a sense of belonging may shape how individuals assess threats and access resources over time (14). Reciprocity—that is, the ability to both give and receive—may be particularly important for self-esteem and social identity, both of which are often threatened by HIV/AIDS-related stigma and social exclusion (15–17).

Previous reviews have examined social capital and mental health in the general older population (18), but none have specifically synthesized evidence for OPLWH. Accordingly, the objectives of this review were to systematically identify and describe studies examining the association between social capital and mental health among OPLWH, to map the operationalization of social capital across included studies by distinguishing social support from broader dimensions, to narratively synthesize the direction and strength of associations between social capital dimensions and mental health outcomes, and to identify gaps in the evidence base and propose directions for future research.

## Methods

### Information source, search strategy and study selection process

This systematic review was conducted, and the results were reported following the PRISMA 2020 checklist (19). The protocol for this systematic review has been registered in PROSPERO (CRD42024537839). Several deviations from the registered protocol became necessary after examining the retrieved evidence, including a shift from meta-analysis to narrative synthesis, the addition of exploratory subgroup descriptions, and the use of the Newcastle-Ottawa Scale instead of the Cochrane Handbook. These deviations are documented in detail in Additional File 3.

All peer-reviewed published articles were systematically searched via several electronic databases, including the Cochrane Library, Web of Science, PubMed, Google Scholar, EMBASE, Scopus, and PsycINFO. We used the following search terms and keywords in the PubMed database: ((((“Social Capital”[Mesh]) OR (((((((((((Social Capital[Title/Abstract]) OR (social cohesion[Title/Abstract])) OR (social networks[Title/Abstract])) OR (social participation[Title/Abstract])) OR (social support[Title/Abstract])) OR (social engagement[Title/Abstract])) OR (reciprocity[Title/Abstract])) OR (social connectedness[Title/Abstract])) OR (social trust[Title/Abstract])) OR (social integration[Title/Abstract])) OR (collective efficacy[Title/Abstract]))) AND ((“Mental Health”[Mesh]) OR (((((((((((Mental Health[Title/Abstract]) OR (psychological health[Title/Abstract])) OR (psychological problem[Title/Abstract])) OR (emotional problem[Title/Abstract])) OR (Mental Disorder[Title/Abstract])) OR (mental Illness[Title/Abstract])) OR (Depression[Title/Abstract])) OR (Anxiety[Title/Abstract])) OR (Hypervigilance[Title/Abstract])) OR (Nervousness[Title/Abstract])) OR (Anxiousness[Title/Abstract])))) AND ((“Aged”[Mesh]) OR ((((Aged[Title/Abstract]) OR (Elderly[Title/Abstract])) OR (Older adults[Title/Abstract])) OR (Senior[Title/Abstract])))) AND ((“HIV”[Mesh]) OR (((HIV[Title/Abstract]) OR (PLWH[Title/Abstract])) OR (AIDS[Title/Abstract]))). For the other six electronic databases (Cochrane Library, Web of Science, Google Scholar, EMBASE, Scopus, and PsycINFO), specific database subject headings linked with the above terms and keywords were used. The search was limited to studies published in English and included publications up to November 28, 2025. Additionally, we manually searched the reference lists of the included studies to identify additional articles. Using EndNote software, the titles, abstracts, duplications, and full-text contents of the identified publications were evaluated against the prespecified inclusion and exclusion criteria.

We employed the PECO (Population, Exposure, Comparator, Outcome) framework to determine eligibility:

(1) Population: older people living with HIV (age ≥50 years);
(2) Exposure: high social capital (operationalized primarily as social support, but also including other dimensions such as social participation, trust, or cohesion when assessed);
(3) Comparator: low social capital/support;
(4) Outcome: mental health status (depressive symptoms, anxiety symptoms, or overall mental health scores).

### Eligibility criteria

#### Inclusion criteria

(1) Observational studies (cohort or cross-sectional) that quantitatively assessed the association between at least one dimension of social capital and a mental health outcome in older people living with HIV.
(2) Study participants were diagnosed with HIV and were ≥50 years of age at the time of the study (or reported subgroup data for this age group), as 50 years is commonly used as the cut-off for “older” in HIV research (20).
(3) Studies had to report an effect size (odds ratio, risk ratio, or unstandardized regression coefficient) with either a 95% confidence interval or exact *p*-value, or provide sufficient data to calculate these.
(4) The search was limited to English-language publications; there were no restrictions on sex, nationality, religion, or ethnicity.

#### Exclusion criteria

(1) Studies such as clinical trials, literature reviews, commentaries, short communications, and letters to the editor.

(2) Studies on the relationship between exposures other than social capital and the mental health of older people living with HIV.

The full details are provided in Additional File 1.

### Definition of outcome variables

Mental health outcomes included both categorical diagnoses and symptom severity scores measured by validated instruments. Categorical diagnoses were based on criteria such as the International Classification of Diseases (ICD) (21) or the Diagnostic and Statistical Manual of Mental Disorders (DSM) (22). Continuous symptom severity was assessed using validated scales, including the Center for Epidemiologic Studies Depression Scale (CES-D-10), Patient Health Questionnaire (PHQ-9), General Health Questionnaire (GHQ-12), Generalized Anxiety Disorder 7-item scale (GAD-7), Symptom Checklist-90 (SCL-90), Beck Depression Inventory (BDI/BDI-II), Center for Epidemiologic Studies Depression Scale Revised (CESD-R), and the mental health summary score of the Short Form-12 (SF-12 MCS) and RAND-36 emotional quality of life. Outcomes were grouped by construct (depressive symptoms, anxiety, overall mental health/well-being, and psychological distress) for narrative synthesis.

### Definition of the exposure variable

Social capital is a multidimensional construct encompassing social support, trust, reciprocity, civic participation, and social cohesion (7–11). In this review, the exposure was defined as any dimension of social capital measured in relation to a mental health outcome. Although the majority of included studies measured social support, either as a global construct or through specific subtypes (emotional, instrumental, perceived), the measurement instruments varied and included the Medical Outcomes Study (MOS) Social Support Survey, the Perceived Social Support Scale (PSSS), the ENRICHD Social Support Instrument (ESSI), a 10-item social support questionnaire, the Duke Social Support Index, the Social Relationship Scale, and the Social Network Index (23–30). Two studies used a broader health-related social capital instrument distinguishing individual/family and community/society dimensions (6, 31). We therefore mapped the dimensions measured across studies (Table 1) and report findings by dimension, while acknowledging that social support was the most frequently assessed component of social capital.

**Table 1:** Social capital dimensions captured by the measurement instruments in included studies^a^.

Dimension	Number of studies measuring (*n*/*N*)	Studies	Notes
Social support (overall or specific)	9/10	Han et al. (6); Kapadia et al. (23); Yang et al. (24); Yeji et al. (25); Chesney et al. (26); Mavandadi et al. (27); Whitehead et al. (28); Sun et al. (29); Lin et al. (31)	Most commonly measured dimension; includes perceived, received, emotional, instrumental, tangible, and positive social interaction.
Social network size/structure	1/10	Yoo-Jeong et al. (30)	Measured as diversity of social ties (Social Network Index).
Individual/family social capital	2/10	Han et al. (6); Lin et al. (31)	Includes support from spouse/relatives/close contacts, trust, and satisfaction with marriage (IF scale).
Community/society social capital	2/10	Han et al. (6); Lin et al. (31)	Includes participation, support from community organizations, trust in health/government, and perceived fairness (CS scale).
Trust	2/10	Han et al. (6); Lin et al. (31) (as part of CS scale)	Not assessed as a standalone construct.
Civic participation	2/10	Han et al. (6); Lin et al. (31) (single item)	Very low endorsement; not analyzed separately.
Reciprocity	0/10	—	Not measured.
Bonding/bridging/linking	0/10	—	Not measured.

^a^Counts indicate whether the dimension was captured by any instrument used in the study, regardless of whether it was analysed as a standalone exposure. Trust and civic participation were captured only as part of a broader community/society social capital scale and were not analysed separately.

### Quality appraisal and methods of data extraction

Methodological quality was assessed using the Newcastle-Ottawa Scale (NOS). For the nine cross-sectional studies, the adapted version for cross-sectional studies (NOS-xs) was employed, which evaluates study sample selection (max 2 stars), assessment of exposure(s) and outcome(s) (max 4 stars), and confounding factors (max 3 stars), yielding a maximum score of 9. Scores of 7–9 were considered low risk of bias, 4–6 moderate, and 0–3 high. The one cohort study (6) was assessed using the standard NOS for cohort studies (max 9 stars) (32, 33). Two reviewers independently assessed methodological quality, and any disagreements were resolved through discussion with the review team until consensus was reached. Data extraction was performed via a specific format prepared in an Excel spreadsheet. The following information was extracted from eligible full-text articles: author name, year of publication, country, sample size, study design, type of support, instrument employed, and measures of association and confidence intervals. The adjustment variables included in each original model were also extracted and are summarized in Additional File 4.

### Synthesis approach

A narrative approach was used to collate and summarise the breadth of social capital dimensions examined and to describe the characteristics of the published literature. All included studies were summarised and described according to study characteristics. Summarising the studies in this manner allowed us to determine the most appropriate data synthesis method. In the absence of prespecified criteria for deciding when to conduct a meta-analysis, we assessed the suitability of meta-analysis based on established methodological guidance (34, 35). Given the limited number of eligible studies and expected heterogeneity in study design, populations, definitions of social capital, number and types of dimensions assessed, mental health outcomes and instruments, and methods of exposure and outcome ascertainment, meta-analysis was ultimately considered inappropriate, as it would not have provided a meaningful or interpretable synthesis of the available evidence (34, 36). We used the guidelines for synthesis without meta-analysis to conduct the narrative review (34, 36). We reported the direction of association (inverse, positive, or null) and statistical significance as the primary summary measures. We also constructed an evidence map to visualize which dimensions and outcomes have been examined.

## Results

### Selection of studies

As shown in Figure 1, a total of 9,060 papers were retrieved from seven electronic databases during the search. After the initial screening of article titles and abstracts, 358 papers remained. After removing duplicates and screening full texts, 10 articles that met the inclusion criteria were included in this systematic review. For studies where data were not accessible in the original article, the authors were contacted via email to obtain the necessary data.

**Figure 1:**
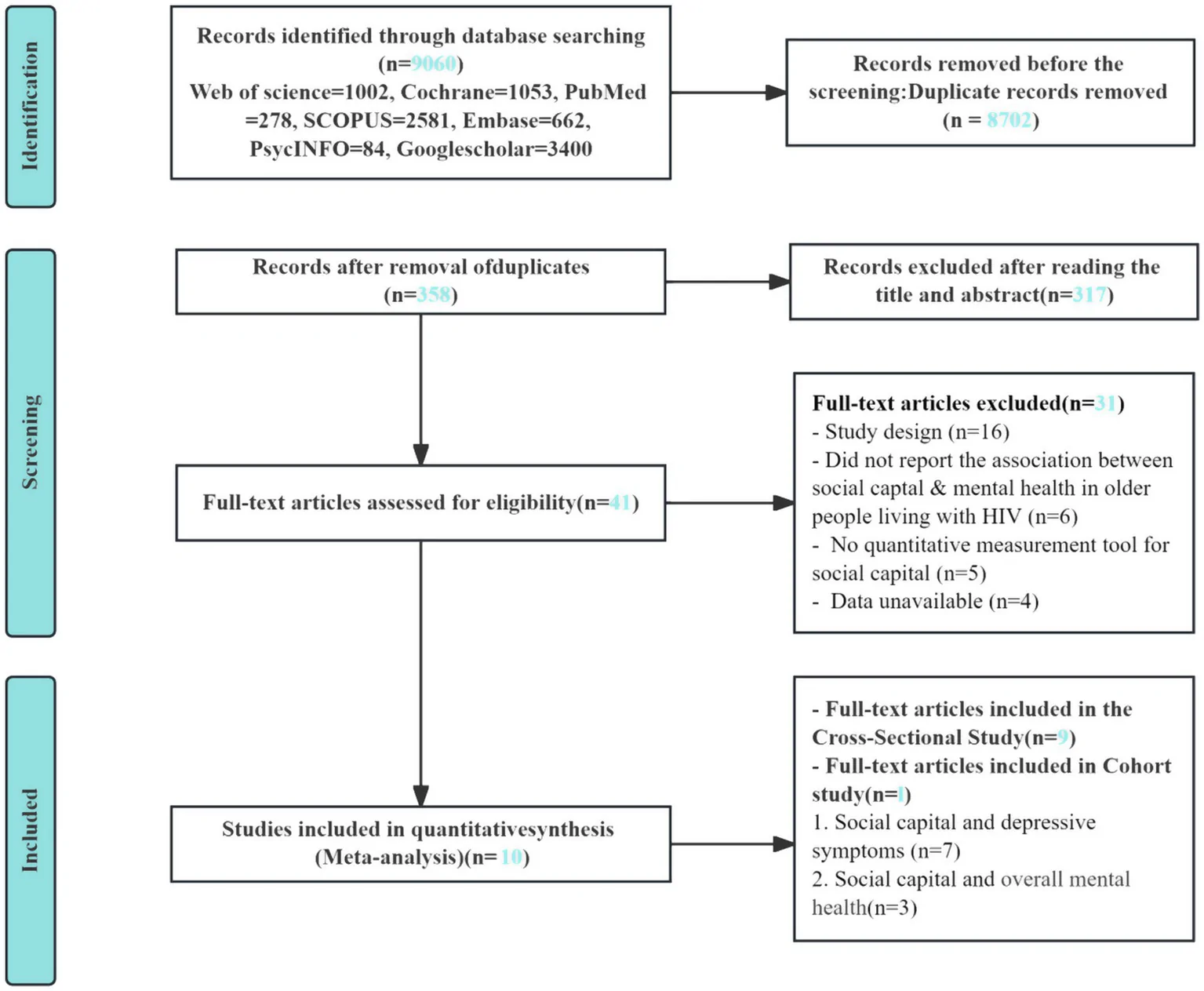
PRISMA flow chart of the study identification process for systematic reviews.

### Characteristics of included studies

Table 2 summarizes the characteristics of the 10 included studies. Studies were conducted in the United States (*n* = 5), China (*n* = 4), and South Africa (*n* = 1). Sample sizes ranged from 35 to 529 participants. Nine studies were cross-sectional, and one was a cohort baseline analysis (6). Studies were eligible if they enrolled adults aged ≥50 years or reported subgroup data for adults aged ≥50 years. Publication years ranged from 2003 to 2023.

**Table 2:** Summary characteristics of studies investigating the association between social capital and mental health among older people living with HIV (*N* = 10, 2003–2023).

Number	Author, year, setting	Type of study	Sample size	Type of social capital	Measurement		Measure of association	Quality score^a^
					Social capital	Mental health		
1	Yang et al. (24), 2015, China	Cross-sectional study	120	Positive social interaction	MOS-SSS	Depression: CES-D-10	*t* = −3.39 *p* < 0.001	7/9
2	Yeji et al. (25), 2014, South Africa	Cross-Sectional study	272	emotional support and instrumental support	A 10-item social support questionnaire	Depression: GHQ-12	AOR = 0.65 (0.52, 0.81) *p* < 0.001	7/9
3	Han et al. (6), 2020, Sichuan, China	Cohort study	529	Individual and family social capital scale (IF)/community and society social capital scale (CS)	A validated Chinese version of health-related social capital measurement	Depression: CES-D-10	IF: OR = 0.89 (0.84, 0.93) *p* < 0.001	8/9
							CS: OR = 0.91 (0.84, 0.99) *p* < 0.05	
						Anxiety: GAD-7	IF: OR = 0.90 (0.86, 0.95), *p* < 0.001	
							CS: OR = 0.96 (0.89, 1.04), *p* > 0.05	
4	Lin et al. (31), 2020, China	Cross-Sectional Study	529	Individual and family social capital scale (IF)/community and society social capital scale (CS)	A validated Chinese version of health-related social capital measurement	Overall Mental health: SF-12	IF: *B* = 0.61 (0.42, 0.81), *p* < 0.001	7/9
							CS: *B* = 0.74 (0.42, 1.06), *p* < 0.001	
5	Kapadia et al. (23), 2016, US	Cross-sectional study	100	Overall functional social support	MOS-SSS	Overall mental health: MINI 6.0	AOR = 0.52 (0.32, 0.84), *p* < 0.05	4/9
6	Sun et al., (29), 2007, China	Cross-sectional study	94	General social support	PSSS	Mental health: SCL-90	*r* = 0.058 *p* > 0.05	4/9
7	Mavandadi et al. (27), 2009, US	Cross-sectional study	35	General social support	An abbreviated version of the Duke Social Support Index	Depression: PHQ-9	*B* = −0.77 *p* < 0.001	7/9
8	Yoo-Jeong et al. (30), 2023, US	Cross-sectional study	146	Social network size/diversity	Social capital: Social Network Index (SNI)	Depression: CESD-R	*B* = −0.531 *p* < 0.001	7/9
						Overall Mental health: RAND-36 emotional QoL	*β* = 0.168, *p* = 0.026	
9	Chesney et al. (26), 2003, US	Cross-sectional study	199	General social support	Social Relationship Scale	Depression: BDI	*B* = −0.420 *p* < 0.01	5/9
10	Whitehead et al. (28), 2014, US	Cross-sectional study	95	Perceived social support	ESSI	Depression: BDI-II	AOR = 0.75 (0.6–0.9), *p* < 0.05	5/9

a NOS-xs for cross-sectional studies, maximum 9; standard NOS for cohort (Han et al.) maximum 9. Scores of 7–9 indicate low risk of bias, 4–6 moderate risk, and 0–3 high risk. Detailed item-level ratings are provided in Additional File 2. The adjustment variables used in each study varied and are detailed in Additional File 4.

### Quality appraisal

Quality scores ranged from 4 to 8 out of 9. Six studies were rated as low risk of bias (score 7–8) and four as moderate risk (score 4–5). No study was classified as high risk of bias. Common methodological limitations included cross-sectional designs, self-reported exposure and outcome measures, incomplete adjustment for confounders, and small sample sizes in some studies. No study was excluded based on quality. Detailed ratings for each item are provided in Additional File 2.

### Operationalization of social capital: a limited evidence base

A key finding of this review is that the operationalization of social capital across studies is limited. Table 1 maps each study to the various dimensions of social capital. Nine of the ten studies measured some form of social support, either as an overall construct or through specific subtypes such as emotional support, instrumental support, or perceived support. Only two studies—Han et al. (6) and Lin et al. (31) (both from the same cohort)—used scales specifically designed to capture broader social capital and distinguished between individual/family-level and community/societal-level dimensions. Even in these studies, measurements focused primarily on perceived support, trust, and participation, without explicitly assessing reciprocity or bonding capital.

No study measured “bridging capital” or “bonding capital” as independent concepts. In the Sichuan Older HIV-Infected Cohort Study (SOHICS) study, trust was partially reflected in the community/societal-level social capital scale (e.g., trust in health and government organizations) but was not treated as a separate dimension. Civic engagement was assessed solely through a single indicator in the SOHICS study, and the response rate for that indicator was extremely low. No study measured reciprocity. Consequently, the evidence base regarding social capital beyond social support is relatively weak. Tables 1, 3 use different counting rules. Table 1 maps whether a dimension was captured by any measurement instrument, whereas Table 3 maps whether that dimension was analysed as an independent exposure in relation to a mental health outcome. Thus, trust and civic participation appear in Table 1 but not in Table 3.

**Table 3:** Number of studies by social capital dimension and mental health outcome^a^.

Social capital dimension	Depressive symptoms	Anxiety	Overall mental health/well-being	Psychological distress
Social support (overall or specific)	8 studies: 7 inverse, 1 null	2 studies: 1 inverse, 1 null trend	3 studies: all positive	2 studies: 1 inverse, 1 null
Social network size/structure	0	0	1 study: positive	0
Individual/family social capital	1 study: inverse	1 study: inverse	1 study: positive	0
Community/society social capital	1 study: inverse	1 study: null	1 study: positive	0
Trust	0	0	0	0
Reciprocity	0	0	0	0
Civic participation	0	0	0	0
Bonding/bridging/linking	0	0	0	0

^a^Counts indicate whether the dimension was analysed as an independent exposure with a mental health outcome. Dimensions captured only within a composite scale or not analysed separately are counted as 0. Therefore, trust and civic participation are not counted in this table.

Table 3 presents an evidence map that cross-analyzes the dimensions of social capital with mental health outcomes. Each cell shows the number of studies and the direction of the association. The map indicates that the relationship between social support and depression, anxiety, overall mental health, and psychological distress has been studied, while other dimensions have been studied rarely or not at all. Next, we will discuss the existing research on the relationship between social capital and the mental health of older people living with HIV, organized by dimension.

### Social support and depression

Eight studies examined the association between social support and depressive symptoms. Most studies reported a negative correlation, meaning that higher levels of social support were associated with milder depressive symptoms. Yang et al. (24) found that positive social interaction—a form of implicit support that can be obtained without disclosing one’s problems—significantly predicted a reduction in depressive symptoms among people living with HIV in China, whereas explicit support dimensions such as emotional/informational support and material support did not show a significant effect. Yeji et al. (25) found that among South African adults receiving antiretroviral therapy, instrumental social support was associated with a reduced risk of depression, whereas emotional support showed no such association. Chesney et al. (26) reported a significant negative correlation between overall social support and a composite measure of psychological distress, as well as a positive correlation with positive emotions. Kapadia et al. (23) found that both higher emotional support and higher positive social support were associated with a reduced likelihood of meeting diagnostic criteria for any mental health condition. Mavandadi et al. (27) reported that higher overall social support was associated with milder depressive symptoms. Whitehead et al. (28) found that higher perceived social support was associated with a reduced likelihood of depression. Two studies from the Chinese SOHICS cohort further corroborate these patterns: Han et al. (6) found that individual/family social capital was negatively associated with probable depression and probable anxiety, while community/societal social capital was associated with depression but not with anxiety; Lin et al. (31) found that both individual/family social capital and community/societal social capital were positively associated with the mental health total score on the SF-12 scale.

However, not all studies reported significant associations. Sun et al. (29) found that, although participants reported moderately high levels of support, there was no significant correlation between perceived social support and the SCL-90 depression subscale. Overall, the direction of the associations is consistent—that is, higher levels of social support or social capital are associated with better mental health—but the strength and statistical significance of the associations vary by dimension and outcome, and no studies have reported harmful associations.

### Social support and anxiety

Only two studies specifically examined anxiety as an outcome. Han et al. (6) found that individual/family social capital was negatively correlated with anxiety, whereas community/societal social capital showed no such correlation. Yang et al. (24) found that positive social interactions were associated with a trend toward lower anxiety levels, though this did not reach statistical significance. Therefore, the evidence regarding anxiety is limited, and the findings are inconsistent.

### Social support and overall mental health, well-being, or general mental health status

Several studies assessed broader mental health outcomes using different measures. Lin et al. (31) found positive correlations between individual/family and community/society social capital and the SF-12 mental health summary score. Yoo-Jeong et al. (30) found that social network size predicted emotional quality of life but did not predict physical or social quality of life. Yang et al. (24) reported that positive social interactions were associated with better overall mental health scores and quality of life, although these associations were only marginally significant. Chesney et al. (26) found that social support was associated with positive emotions. In addition, Kapadia et al. (23) found that higher emotional support and higher positive social support were each associated with lower odds of meeting criteria for any mental health condition. These studies consistently indicate that social resources are associated with better mental health, but there are differences in the conceptualization of “overall mental health,” and the number of studies is limited.

### Social capital at the community/societal level

Only two SOHICS studies measured social capital at the community/societal level. Han et al. (6) found a weak negative correlation with depression but no significant association with anxiety. Lin et al. (31), on the other hand, found a positive correlation with the composite mental health score. It is worth noting that participation at the community level was extremely low in this Chinese cohort study, and the measurement instrument primarily reflected trust in institutions and perceptions of fairness. Therefore, the evidence regarding the impact of community-level social capital on people living with HIV and AIDS is extremely limited and context-specific.

### Unmeasured dimensions: trust, reciprocity, participation, bonding capital/bridging capital/linking capital

No studies have examined reciprocity, bonding capital, bridging capital, or linking capital. Trust is partially reflected in the Community/Social subscale of the SOHICS, but it has not been analyzed separately. Civic engagement was assessed through only one item, and the item’s reliability was extremely low. This indicates a research gap: it remains unclear whether these dimensions have unique or independent associations with the mental health of OPLWH.

## Discussion

This systematic review reveals a mismatch between the breadth of the concept of social capital and the ways in which it has been operationalized in studies of older people living with HIV. Although social capital theoretically encompasses trust, reciprocity, participation, and multidimensional network resources, the existing evidence is primarily dominated by measures of perceived or received social support.

The findings in this review—that higher levels of social support are typically associated with better mental health—are consistent with the broader literature on older people (16, 37, 38). However, the strength of this conclusion is constrained by several limitations. Nearly all studies were cross-sectional in design, making it impossible to draw inferences about causality. It is just as plausible that mental health problems may erode social networks as it is that weak social networks may worsen mental health. Measurements of social support and mental health varied widely, making direct comparisons difficult, a limitation that has also been observed in systematic reviews of social capital and mental well-being in the general older population (18, 39). The few studies that examined dimensions beyond social support were based on the same Chinese cohort, which limits generalizability.

Perhaps the most significant contribution of this review is that it identifies areas that have not yet been studied. Reciprocity, civic engagement, trust as an independent construct, and bonding, bridging, and linking capital have been almost entirely overlooked. This is important for both theory and practice. Older people living with HIV may derive psychological benefits not only from receiving support, but also from contributing to others, participating in community life, or feeling that institutions are trustworthy. These mechanisms may be particularly important in the context of HIV-related stigma and social isolation (40, 41). Without measuring these dimensions, we cannot know whether interventions should focus on enhancing the support received, fostering reciprocal relationships, building community trust, or promoting civic engagement.

The evidence map also shows that outcomes other than depressive symptoms are rarely examined. Anxiety, positive emotions, and mental health-related quality of life have been assessed in only a few studies, and no studies have examined post-traumatic stress or complex psychological outcomes, despite their documented relevance to aging with HIV (42). Our findings are consistent with previous systematic reviews in related fields. A 2017 systematic review of social capital interventions targeting older people found some evidence that such interventions, primarily those enhancing social support, may improve well-being and reduce loneliness, although the quality of evidence was limited (18). Similarly, a systematic review on social capital and mental illness among the general adult population reported an inverse association but also noted the same conceptual and measurement heterogeneity that we observed (14). This review expands upon the existing literature by specifically focusing on older people living with HIV, a population that may face unique challenges in establishing and maintaining social capital due to stigma, concerns about disclosure, and the cumulative loss of peers.

These gaps offer specific implications for future research. Studies should employ multidimensional social capital measures that, in addition to support, explicitly assess trust, reciprocity, participation, and network resources. Researchers should distinguish between social capital at the individual/family level and the community/societal level, and include outcomes beyond depression, such as anxiety, loneliness, positive well-being, and trauma-related symptoms. Longitudinal designs are needed to examine temporal relationships and potential bidirectional effects. Researchers should also report full effect estimates, confidence intervals, and exact *p*-values, as well as results stratified by age and gender, to facilitate future meta-analyses and subgroup comparisons.

For practice and policy, the available evidence suggests that strengthening social support may be a promising direction for supporting mental health in OPLWH, but it is premature to recommend specific community-based social capital programs as evidence-based interventions. Healthcare providers should continue to assess social support and social isolation as part of comprehensive care for OPLWH, while recognizing that the evidence base does not yet establish causal benefits. Policies aimed at reducing stigma and promoting social inclusion may plausibly improve mental health, but rigorous evaluation is needed before such programs can be recommended with confidence.

This review has several limitations. The small number of studies and their heterogeneity made it impossible to conduct a meta-analysis. The restriction to English-language studies may have excluded relevant studies in other languages. The quality of the included studies was moderate, with most relying on self-reported measures and cross-sectional designs.

## Conclusion

This systematic review identified only 10 eligible studies, with substantial conceptual and methodological heterogeneity limiting the comparability and generalizability of findings. Research on social capital and mental health in older people living with HIV has concentrated overwhelmingly on social support, leaving broader dimensions of social capital—including trust, reciprocity, civic participation, and bonding/bridging/linking capital—largely unexamined. Higher social support is generally associated with better mental health, but the evidence is cross-sectional and does not support causal conclusions. The principal contribution of this review is to highlight the substantial gap between the theoretical construct of social capital and its empirical operationalization. Future studies should adopt standardized, multidimensional social capital measures and longitudinal designs to clarify directionality.

## References

[1] Hsieh E Polo R Qian HZ Fuster-RuizdeApodaca MJ Del Amo J. Intersectionality of stigmas and health-related quality of life in people ageing with HIV in China, Europe, and Latin America. Lancet Healthy Longev. (2022) 3:e206–15. doi: 10.1016/S2666-7568(22)00003-4 36098292

[2] Fu L Tian T Wang B Lu Z Bian J Zhang W . Global, regional, and national burden of HIV and other sexually transmitted infections in older adults aged 60–89 years from 1990 to 2019: results from the global burden of disease study 2019. Lancet Healthy Longev. (2024) 5:e17–30. doi: 10.1016/S2666-7568(23)00214-3 38183996

[3] Rueda S Law S Rourke SB. Psychosocial, mental health, and behavioral issues of aging with HIV. Curr Opin HIV AIDS. (2014) 9:325–31. doi: 10.1097/COH.0000000000000071, 24824890

[4] Ahmed GY Saha C Almusalami EM Rabaan AA Alhumaid S Ali AA . Prevalence of depression in elderly people living with HIV: a systematic review and meta-analysis. Infect Microbes Dis. (2023) 5:167–71. doi: 10.1097/IM9.0000000000000132

[5] do Carmo Filho A Fakoury MK Eyer-Silva WA Neves-Motta R Kalil RS Ferry FRA. Factors associated with a diagnosis of major depression among HIV-infected elderly patients. Rev Soc Bras Med Trop. (2013) 46:352–4. doi: 10.1590/0037-8682-1228-2013, 23856860

[6] Han J Jia P Huang Y Gao B Yu B Yang S . Association between social capital and mental health among older people living with HIV: the Sichuan older HIV-infected cohort study (SOHICS). BMC Public Health. (2020) 20:581. doi: 10.1186/s12889-020-08705-6 PMC7189431 32345273

[7] Kawachi I Berkman LF. "Social capital, social cohesion, and health". In: Berkman LF Kawachi I Glymour MM, editors. Social Epidemiology, 2nd Edn. New York: Oxford University Press (2014). p. 290–319.

[8] Ehsan AM De Silva MJ. Social capital and common mental disorder: a systematic review. J Epidemiol Community Health. (2015) 69:1021–8. doi: 10.1136/jech-2015-205868, 26179447

[9] Bourdieu P. "Ökonomisches Kapital – Kulturelles Kapital – Soziales Kapital". In: Kreckel R, editor. Soziale Ungleichheiten. Göttingen: Vandenhoeck & Ruprecht (1983). p. 183–98.

[10] Putnam RD. Bowling Alone: The Collapse and Revival of American Community. New York: Simon & Schuster (2001).

[11] Uphoff N. "Understanding social capital: learning from the analysis and experience of participation". In: Dasgupta P Serageldin I, editors. Social Capital: A Multifaceted Perspective. Washington (DC): World Bank (2000)

[12] Woolcock M. Social capital and economic development: towards a theoretical synthesis and policy framework. Theory Soc. (1998) 27:151–208. doi: 10.1023/A:1006884930135

[13] Harpham T Grant E Thomas E. Measuring social capital within health surveys: key issues. Health Policy Plan. (2002) 17:106–11. doi: 10.1093/heapol/17.1.106, 11861592

[14] De Silva MJ McKenzie K Harpham T Huttly SRA. Social capital and mental illness: a systematic review. J Epidemiol Community Health. (2005) 59:619–27. doi: 10.1136/jech.2004.029678, 16020636 PMC1733100

[15] Moore S Kawachi I. Twenty years of social capital and health research: a glossary. J Epidemiol Community Health. (2017) 71:513–7. doi: 10.1136/jech-2016-208313, 28087811

[16] Berkman LF Glass T Brissette I Seeman TE. From social integration to health: Durkheim in the new millennium. Soc Sci Med. (2000) 51:843–57. doi: 10.1016/S0277-9536(00)00065-4, 10972429

[17] Richardson J Postmes T Stroebe K. Social capital, identification and support: scope for integration. PLoS One. (2022) 17:e0266499. doi: 10.1371/journal.pone.0266499, 35421128 PMC9009600

[18] Coll-Planas L Nyqvist F Puig T Urrútia G Solà I Monteserín R. Social capital interventions targeting older people and their impact on health: a systematic review. J Epidemiol Community Health. (2017) 71:663–72. doi: 10.1136/jech-2016-208131, 27834223

[19] Page MJ McKenzie JE Bossuyt PM Boutron I Hoffmann TC Mulrow CD . The PRISMA 2020 statement: an updated guideline for reporting systematic reviews. BMJ. (2021) 372:n71. doi: 10.1136/bmj.n71, 33782057 PMC8005924

[20] The Lancet Healthy Longevity. Ageing with HIV. Lancet Healthy Longev. (2022) 3:e119. doi: 10.1016/S2666-7568(22)00041-1 36098283

[21] World Health Organization. The ICD-10 Classification of Mental and Behavioural Disorders: Clinical Descriptions and Diagnostic Guidelines. Geneva: World Health Organization (1992).

[22] American Psychiatric Association. Diagnostic and Statistical Manual of Mental Disorders. 5th ed. Arlington: American Psychiatric Publishing (2013).

[23] Kapadia F Levy M Rivera A Ransome Y Halkitis PN. Perceived social support and mental health states in a clinic-based sample of older HIV positive adults. J HIV AIDS Soc Serv. (2016) 15:417–27. doi: 10.1080/15381501.2016.1212295

[24] Yang JP Leu J Simoni JM Chen WT Shiu CS Zhao H. "Please don't make me ask for help": implicit social support and mental health in Chinese individuals living with HIV. AIDS Behav. (2015) 19:1501–9. doi: 10.1007/s10461-015-1041-y, 25801475 PMC4526324

[25] Yeji F Klipstein-Grobusch K Newell ML Hirschhorn LR Hosegood V Bärnighausen T. Are social support and HIV coping strategies associated with lower depression in adults on antiretroviral treatment? Evidence from rural KwaZulu-Natal, South Africa. AIDS Care. (2014) 26:1482–9. doi: 10.1080/09540121.2014.931561, 24991994

[26] Chesney MA Chambers DB Taylor JM Johnson LM Folkman S. Social support, distress, and well-being in older men living with HIV infection. J Acquir Immune Defic Syndr. (2003) 33:S185–93. doi: 10.1097/00126334-200306012-00016 12853868

[27] Mavandadi S Zanjani F Ten Have TR Oslin DW. Psychological well-being among individuals aging with HIV: the value of social relationships. J Acquir Immune Defic Syndr. (2009) 51:91–8. doi: 10.1097/QAI.0b013e318199069b, 19282781 PMC2745731

[28] Whitehead NE Hearn LE Burrell L. The association between depressive symptoms, anger, and perceived support resources among underserved older HIV positive black/African American adults. AIDS Patient Care STDS. (2014) 28:507–12. doi: 10.1089/apc.2014.0126 PMC4135320 25090247

[29] Sun H Zhang J Fu X. Psychological status, coping, and social support of people living with HIV/AIDS in Central China. Public Health Nurs. (2007) 24:132–40. doi: 10.1111/j.1525-1446.2007.00617.x, 17319885

[30] Yoo-Jeong M Waldrop-Valverde D McCoy K Ofotokun I. Social network size and its relationship to domains of quality-of-life among older persons living with HIV. AIDS Care. (2023) 35:600–7. doi: 10.1080/09540121.2022.2057907, 35357192 PMC9522889

[31] Lin P Yu B Han J Wang Z Jia P Yang S. Social capital in old people living with HIV is associated with quality of life: a cross-sectional study in China. Biomed Res Int. (2020) 2020:7294574. doi: 10.1155/2020/7294574, 33313316 PMC7721488

[32] Carra MC Romandini P Romandini M. Risk of bias evaluation of cross-sectional studies: adaptation of the Newcastle-Ottawa scale. J Periodontal Res. (2025). doi: 10.1111/jre.13405 [Epub ahead of print]. PMC13665615 40293188

[33] Moskalewicz A Oremus M. No clear choice between Newcastle-Ottawa scale and appraisal tool for cross-sectional studies to assess methodological quality in cross-sectional studies of health-related quality of life and breast cancer. J Clin Epidemiol. (2020) 120:94–103. doi: 10.1016/j.jclinepi.2019.12.013, 31866469

[34] Lensen S. When to pool data in a meta-analysis (and when not to)? Fertil Steril. (2023) 119:902–3. doi: 10.1016/j.fertnstert.2023.03.015, 36948444

[35] Deeks JJ Higgins JPT Altman DG McKenzie JE Veroniki AA. Chapter 10: analysing data and undertaking meta-analyses (last updated November 2024). In: Higgins JP Thomas J. Chandler J Cumpston M Li T Page MJ ., editors. Cochrane Handbook for Systematic Reviews of Interventions Version 6.5. London, United Kingdom: Cochrane (2024). Available online at: https://www.cochrane.org/handbook (Accessed September 24, 2026).

[36] Campbell M McKenzie JE Sowden A Katikireddi SV Brennan SE Ellis S . Synthesis without meta-analysis (SWiM) in systematic reviews: reporting guideline. BMJ. (2020) 368:l6890. doi: 10.1136/bmj.l6890, 31948937 PMC7190266

[37] Wang L Xie S Hu X Li J He S Gao J . Social capital, depressive symptomatology, and frailty among older adults in the western areas of China. PLoS One. (2023) 18:e0292236. doi: 10.1371/journal.pone.0292236, 37788268 PMC10547179

[38] Emlet CA. An examination of the social networks and social isolation in older and younger adults living with HIV/AIDS. Health Soc Work. (2006) 31:299–308. doi: 10.1093/hsw/31.4.299, 17176977

[39] Nyqvist F Forsman AK Giuntoli G Cattan M. Social capital as a resource for mental well-being in older people: a systematic review. Aging Ment Health. (2013) 17:394–410. doi: 10.1080/13607863.2012.742490, 23186534

[40] Brennan-Ing M Seidel L London AS Cahill S Karpiak SE. Social support systems and social network characteristics of older adults with HIV. Interdiscip Top Gerontol Geriatr. (2017) 42:159–72. doi: 10.1159/000448561, 27875831

[41] Stadtler H Weinand S Kohler C Schleussner E Kaestner R Aigner A. Mini-review: elucidating the psychological, physical, and sex-based interactions between HIV infection and stress. Neurosci Lett. (2021) 747:135698. doi: 10.1016/j.neulet.2021.135698 PMC9258904 33540057

[42] Brown MJ Cohen SA DeShields RD. Psychopathology and HIV diagnosis among older adults in the United States: disparities by age, sex, and race/ethnicity. Aging Ment Health. (2020) 24:1746–53. doi: 10.1080/13607863.2019.1636201, 31274001 PMC6942639

