# Supplementary material for: The association between social capital and mental health among older people living with HIV: a systematic review
Source: Front Public Health. 2026 Oct 5;14:1827140. doi: 10.3389/fpubh.2026.1827140 (PMC13679625; doi:10.3389/fpubh.2026.1827140)
Supplement: Supplementary file 1 [file Table_1.DOCX]

Additional file 1 Study inclusion and exclusion criteria.

| Include | Exclude |
| --- | --- |
| Participants |  |
| Adults diagnosed with HIV and aged ≥50 years at the time of the study, or studies reporting subgroup data for this age group. | (1) Participants aged <50 years. (2) Age unclear and no subgroup data for adults aged ≥50 years. |
| Exposure |  |
| Studies that quantitatively assessed at least one dimension of social capital using validated scales. Social capital refers to social resources and networks of relationships that may promote health, including material support (e.g., health facilities and services) and non-material factors such as trust, norms, social support, and social participation. | Studies that did not quantitatively assess social capital. |
| Comparator |  |
| Low social capital/support (as defined under Exposure). |  |
| Outcomes |  |
| Depression, anxiety (diagnosed by a physician, hospital discharge register, or any validated scale with an appropriate cut-off), or mental health summary scores measured by validated scales. | Studies that did not report an association between social capital and mental health. |
| Study design |  |
| Observational studies (cohort, cross-sectional, or case-control) that reported a quantitative association between social capital and mental health (e.g., odds ratio, risk ratio, regression coefficient, or correlation) with confidence intervals or exact p-values, or provided sufficient data to calculate these. | Non-observational studies |

Type of documents: peer-reviewed papers published in academic journals.

Publication date, sex, nationality, religion, and ethnicity were not restricted. The search was limited to English-language publications; no non-English studies were included.
