# Supplementary material for: The association between social capital and mental health among older people living with HIV: a systematic review
Source: Front Public Health. 2026 Oct 5;14:1827140. doi: 10.3389/fpubh.2026.1827140 (PMC13679625; doi:10.3389/fpubh.2026.1827140)
Supplement: Supplementary file 2 [file Table_2.docx]

Additional file 2 Quality assessment scores for studies investigating the association between social capital and mental health in older people living with HIV (N = 10, 2003-2023)

| **Question** | **Score** | | | | | | | | | |
| --- | --- | --- | --- | --- | --- | --- | --- | --- | --- | --- |
|  | **Yang JP., et al**  **2015**  **China** | **Yeji F., et al**  **2014**  **South Africa** | **Han J., et al**  **2020**  **Sichuan，China** | **Lin P., et al**  **2020**  **China** | **Kapadia F., et al**  **2016**  **US** | **Sun H., et al**  **2007**  **China** | **Mavandadi S., et al., 2009 US** | **Yoo-Jeong M., et al**  **2023**  **US** | **Chesney MA., et al**  **2003**  **US** | **Whitehead NE., et al., 2014 US** |
| **STUDY SAMPLE SELECTION**  **(max. 2 stars)** | **1** | **1** | **2** | **1** | **0** | **0** | **0** | **1** | **0** | **0** |
| **ASSESSMENT of EXPOSURE and OUTCOME (max. 4 stars)** | **3** | **3** | **3** | **3** | **3** | **2** | **3** | **3** | **3** | **3** |
| **CONFOUNDING FACTORS**  **(max. 3 stars)** | **2** | **2** | **2** | **2** | **1** | **0** | **0** | **2** | **2** | **2** |
| **Total score**  **(maximum 9)** | **6** | **6** | **7** | **6** | **4** | **2** | **3** | **6** | **5** | **5** |
| **“7–9 stars” represents Low RoB, “4-6 stars” represents Moderate RoB, “0-3 stars” represents High RoB** | | | | | | | | | | |
