# Supplementary material for: The association between social capital and mental health among older people living with HIV: a systematic review
Source: Front Public Health. 2026 Oct 5;14:1827140. doi: 10.3389/fpubh.2026.1827140 (PMC13679625; doi:10.3389/fpubh.2026.1827140)
Supplement: Supplementary file 3 [file Table_3.DOCX]

**Protocol deviations**

This review was registered in PROSPERO (CRD42024537839). Several deviations from the registered protocol became necessary after examining the retrieved evidence and are documented below.

First, the registered synthesis plan specified pooling odds ratios for binary outcomes and weighted mean differences for continuous outcomes, and did not specify conversion of heterogeneous estimates into partial correlations. Because the eligible studies reported heterogeneous effect estimates (odds ratios, adjusted odds ratios, and unstandardized regression coefficients) and because conceptual and measurement heterogeneity was substantial, we did not perform a meta-analysis. Instead, we conducted a narrative synthesis without meta-analysis, consistent with established methodological guidance [27,29]. This change was made to avoid misleading precision from pooling non-comparable estimates. The review title was therefore changed from “a meta-analysis” to “a systematic review”.

Second, the registered protocol stated “Analysis of subgroups or subsets: None.” We subsequently conducted exploratory subgroup descriptions by country income level, sample size, and publication year. These analyses were post hoc and are clearly labeled as exploratory; they were not prespecified and should not be interpreted as confirmatory.

Third, the registered protocol specified that Egger’s test would be conducted only for outcomes including ten or more studies. Because no outcome included ten or more studies (depression: seven studies; overall mental health: three studies), and because we did not perform meta-analysis, Egger’s test and trim-and-fill analysis were not performed. This is consistent with the protocol’s minimum threshold and with concerns about the validity of publication-bias tests when few studies are available.

Fourth, the registered protocol proposed quality assessment using the Cochrane Handbook for Systematic Reviews of Interventions. Because all but one included study were observational (cross-sectional), we used the Newcastle-Ottawa Scale (NOS), including the adapted version for cross-sectional studies (NOS-xs) and the standard NOS for the one cohort study. This tool is more appropriate for observational evidence.

Fifth, the PROSPERO record contains inconsistent statements about language restrictions. The searches were restricted to English-language publications, and all included studies were published in English. No non-English studies were included.

Additional file 3 Deviations from the registered PROSPERO protocol

| **Protocol item** | **Registered plan** | **Actual approach** | **Rationale** |
| --- | --- | --- | --- |
| Review type | Meta-analysis | Systematic review with narrative synthesis | Effect estimates and constructs too heterogeneous to pool; risk of misleading precision |
| Synthesis method | Pool odds ratios for binary outcomes and weighted mean differences for continuous outcomes | Narrative synthesis; no meta-analysis | Heterogeneous effect estimates (ORs, AORs, unstandardized regression coefficients) not comparable |
| Effect size conversion | Not specified | No conversion; original estimates reported | Conversion to partial correlations not defensible for adjusted estimates with incomplete p-values |
| Subgroup analyses | None prespecified | Exploratory descriptions by country income, sample size, publication year | Post hoc; labeled exploratory |
| Publication bias assessment | Egger’s test only if ≥10 studies | Not performed | No outcome had ≥10 studies; meta-analysis not performed |
| Quality assessment tool | Cochrane Handbook | Newcastle-Ottawa Scale (NOS-xs for cross-sectional; NOS for cohort) | All but one study observational; NOS more appropriate |
| Language restriction | Inconsistent statements in PROSPERO record | Searches restricted to English; all included studies in English | Actual practice documented; no non-English studies included |
