# Supplementary material for: The association between social capital and mental health among older people living with HIV: a systematic review
Source: Front Public Health. 2026 Oct 5;14:1827140. doi: 10.3389/fpubh.2026.1827140 (PMC13679625; doi:10.3389/fpubh.2026.1827140)
Supplement: Supplementary file 4 [file Table_4.docx]

Additional file 4 Adjustment variables in included studies

| Study | Adjustment variables |
| --- | --- |
| Yang et al., 2015 | Age, gender, income, education, employment |
| Yeji et al., 2014 | Sex, age, marital status, education, household wealth, CD4 cell count |
| Han et al., 2020 (depression) | Age, sex, education, monthly personal income, route of HIV transmission, time since diagnosis |
| Han et al., 2020 (anxiety) | Age, sex, education, marital status, employment, monthly personal income, route of HIV transmission, time since diagnosis, duration of ART |
| Lin et al., 2020 | Age, sex, marital status, employment, route of HIV transmission, spouse HIV status, time since diagnosis, duration of ART |
| Kapadia et al., 2016 | Gender, race/ethnicity |
| Sun et al., 2007 | None (bivariate correlations only) |
| Mavandadi et al., 2009 | None (no additional covariates adjusted; age group was the primary predictor) |
| Yoo-Jeong et al., 2023 | Sex, past unstable housing, comorbidity burden, functional status, depressive symptoms, HIV stigma, loneliness |
| Chesney et al., 2003 | AIDS diagnosis, disability, HAART |
| Whitehead et al., 2014 | Sex, income, years since HIV diagnosis |
